# Analysis of Risk Factors Associated with the Development of Hepatocellular Carcinoma in Chronic HBV-Infected Chinese: A Meta-Analysis

**DOI:** 10.3390/ijerph13060604

**Published:** 2016-06-17

**Authors:** Xiang Lyu, Kui Liu, Yongdi Chen, Zhifang Wang, Jun Yao, Gaofeng Cai, Zhenggang Jiang, Zhengting Wang, Jianmin Jiang, Hua Gu

**Affiliations:** 1 Department of Statistics, Purdue University, West Lafayette, IN 47907, USA; alanlvruc@gmail.com; 2 School of Statistics, Renmin University of China, Beijing 100872, China; 3 Zhejiang Provincial Center for Disease Control and Prevention, 3399 Binsheng Road, Hangzhou 310051, China; kliu@cdc.zj.cn (K.L.); ydchen@cdc.zj.cn (Y.C.); zfwang@cdc.zj.cn (Zhi.W.); jyao@cdc.zj.cn (J.Y.); gfcai@cdc.zj.cn (G.C.); zhgjiang@cdc.zj.cn (Z.J.); ztwang@cdc.zj.cn (Zhe.W.)

**Keywords:** hepatitis B virus, hepatocellular carcinoma, meta-analysis, risk factor

## Abstract

Hepatitis B virus (HBV) infection is a major risk factor for the development of hepatocellular carcinoma (HCC) in China. At present, there still are 9.3 million chronic HBV-infected Chinese. Numerous studies have explored the association between possible factors and hepatocellular carcinoma risk, however, the results remains inconsistent. Therefore, we did this pooled analysis so as to get a precise result. Here, we took the chronic HBV-infected Chinese as the object. We systematically searched for studies evaluating whether the proposed factors changed HCC risk in PubMed, Chinese National Knowledge Infrastructure, VIP database and Wanfang data. Odds ratios (OR) with 95% confidence intervals (CI) were calculated by Review Manager 5.0 and publication bias was determined by Begg’s test and Egger’s test. In total, 3165 cases and 10,896 controls from 27 studies were included in this meta-analysis. Our results showed that pooled OR with 95% CI for each of the factors investigated were: non-antiviral treatment 2.70 (2.01, 3.62), high HBV DNA levels 2.61 (1.73, 3.94), alcohol consumption 2.19 (1.53, 3.13), a family history of HCC 3.58 (2.53, 5.06) and male gender 2.14 (1.68, 2.73), respectively. Our meta-analysis supports that high HBV DNA levels, non-antiviral treatment, alcohol consumption, a family history of HCC and male gender contributed to the risk of hepatocellular carcinoma in chronic HBV-infected Chinese from currently available evidence. Given the high prevalence of the non-antiviral treatment and alcohol drinking, behavior interventions for the two factors should be tackled first.

## 1. Introduction

Liver cancer, which consists predominantly of HCC, is the fifth most common carcinoma and the third most common cause of tumor-related deaths worldwide, leading to approximately 500,000 deaths every year [1,2,3]. In China, the incidence of HCC is increasing and now accounts for 55% of all HCC cases in the world [4,5]. 

Considering the scale of the public health problem posed by HCC, over the past few decades, some measures have also been introduced to reduce the aflatoxin content in food and to reduce the lyngbya toxins in drinking water in China [6,7,8]. However, the incidence of HCC continues, in which HBV-related HCC patients account for about 80% of all HCC [9].

Since 1992, as part of an immunization project in China, hepatitis B vaccine has been used to inoculate in all newborns to effectively block mother-to-infant HBV transmission. However, the HBV infection rate remains high, with HBsAg carry rate of 7.2% for those aged between 1 and 59 years and 9.3 million chronic HBV-infected Chinese [10,11]. Whereas the 9.3 million chronic HBV-infected Chinese will be the major source of HCC over the next 50 years, though not all the chronic HBV carriers have developed HCC, existing medical interventions are unable to cure chronic HBV-related diseases and only serve to slow disease progression. Hence, our study focused on the 9.3 million chronic HBV-infected Chinese. 

Previous studies showed the modifiable risk factors included persistent high level of HBV replication, smoking and the habitual use of alcohol, however the effects of these factors were controversial [12,13,14,15,16,17,18,19,20,21,22,23,24,25,26,27,28,29,30,31,32,33,34,35,36,37,38,39,40]. Considering the scale of the public health problem accompanied with chronic HBV infection, the major risk factors to the development of HCC in chronic HBV-infected Chinese were investigated, an understanding of which is vital to block the development to HCC by effective prevention and control measures. 

Meta-analysis is the implementation of statistical methods for combining and contrasting results from different studies to reduce random error and identify patterns or relationships in the context of a variety of evidence. In this study, we performed this meta-analysis to identify the true associations between possible factors and HCC risk.

## 2. Materials and Methods

### 2.1. Ethics 

The data reported in our manuscript were cited from published literature, and had been approved by the ethics committee of Zhejiang provincial center of disease control and prevention (No.zjcdc-2015-1).

### 2.2. Literature and Search Strategy

All articles were retrieved from the following databases: Pubmed, Chinese National Knowledge Infrastructure (CNKI), VIP database and Wanfang data. Searches, using the search field “Title/Abstract”, were performed via using the search terms (“Chinese” or “China”) and (“hepatocellular carcinoma” or “liver tumors” or “tumor of liver” or “liver neoplasms”) and (“hepatitis b” or “hepatitis b virus”) AND (“risk factor”) from PubMed. Searches, using the search field “Abstract”, were performed via using the above-mentioned search terms from CNKI, VIP and Wanfang data journals published between January 1980 and October 2014.

The present study was carried out following Meta-analysis in PRISMA guidelines [41].

### 2.3. Inclusion and Exclusion Criteria

Only primary studies were included in our search. All eligible articles were case-control or prospective studies between January 1980 and October 2014. Eligible research articles not captured by the research strategies detailed above were retrieved by bibliography searches.

Studies were included in the meta-analysis provided that: (1) the article reported a case-control or prospective study and had been accepted for publication with full text available; (2) all cases and controls were diagnosed by histopathological biopsy, or other national diagnostic criteria existing at that time, and possible risk factors were reported; (3) the article reported on chronic HBV-infected Chinese population (HBsAg as a marker of chronic HBV infection); and (4) the data to calculate OR with 95% CI was reported.

Studies were excluded from the meta-analysis when: (1) The article reported other forms of viral hepatitis (hepatitis C or D) as the etiological agent; (2) The article did not provide a workable value for the main variable.

### 2.4. Data Extraction

To decide whether an article should be included or excluded, two independent reviewers carried out an assessment using a standardized data extraction form designed by our group. Data were extracted from each study by two separate investigators, and data about aflatoxin were not extracted because meta-analysis in the aflatoxin has been done [42]. The literature referenced in the articles included in this study was also screened to identify more studies.

Discrepancies between the decisions of the two reviewers were discussed. If a consensus was not achieved, the decision was made by a third reviewer. Articles were examined to eliminate duplicate reports of the same research.

The following information was extracted from all of the acquired studies: the numbers of patients in each group, the characteristics of each group at baseline (including female/male ratio, average and median ages), and the study type. Definition of main outcomes: HCC, liver cirrhosis, CHB chronic hepatitis B and chronic HBV carries were diagnosed by the guidelines at that time [43,44].

### 2.5. Statistical Analysis

The OR with 95% CI was used as the main outcomes to measure efficacy. Meta-analysis was performed using either the fixed-effect or random-effect model, depending on the statistical heterogeneity among studies as evaluated by Cochran’s chi-square test [45]. Statistical heterogeneity among studies was assessed using the Q and I2 statistics. When *p* ≤ 0.1 the random-effect model was employed, and when *p* > 0.1 the fixed effects model was employed. In this meta-analysis, subgroup analyses were used to more thoroughly investigate the associations between different risk factors and HBV-related HCC, and Begg’s test and Egger’s linear regression test were also used to examine publication bias [46,47]. The results of the Egger’s test indicated that the publication difference of a positive result and a negative result was not statistically significant (all *p* > 0.10), and the results of the Begg’s test were also not statistically significant (all *p* > 0.10). Analyses were performed using the software Stata version 9.0 (Stata Corp., College Station, TX, USA) and Review Manager 5.0 (Cochrane Collaboration, Rigshospitalet, Denmark). The OR was not pooled when the number of OR of the risk factor were less than 5. All of the P-values were two-sided.

## 3. Results

### 3.1. Literature Search

**Figure 1:**
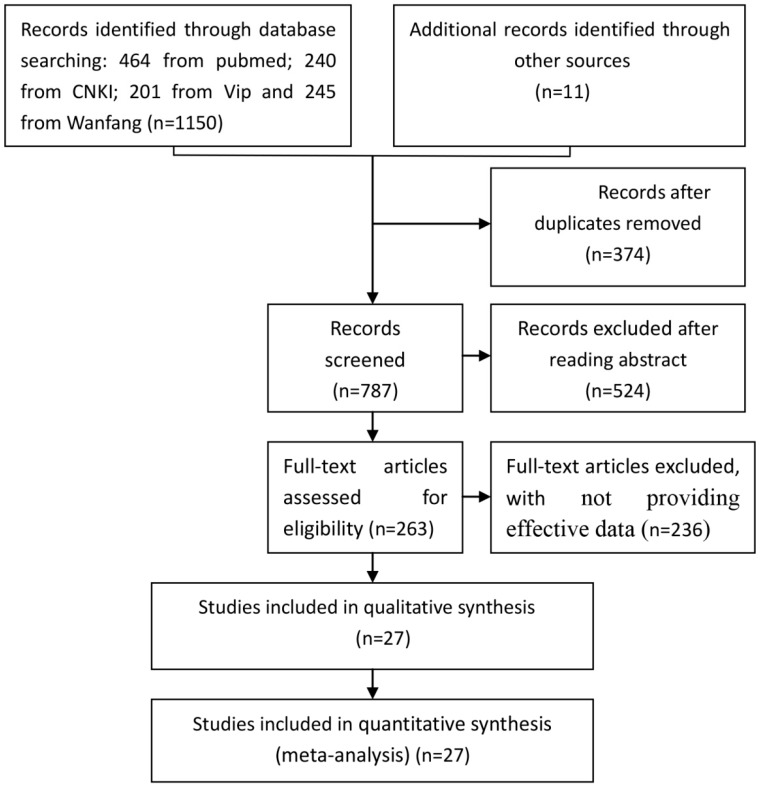
Flowchart of the selection of studies for inclusion in the meta-analysis Abbreviations: CNKI: Chinese National Knowledge Infrastructure; Vip: vip database; Wanfang: Wanfang data; and HCC: hepatocellular carcinoma.

The selection of studies for inclusion in the meta-analysis was shown in Figure 1. 

According to the inclusive and exclusive criteria, all articles were retrieved and carefully reviewed to assess the eligibility. Twenty-seven eligible studies were identified after screening of 1161. 

### 3.2. Characteristics of the Studies

A total of 27 studies, including 3165 cases and 10,896 controls were included in our research. Among the twenty-seven studies, following categories were studied: alcohol consumption (2180 cases), smoking (1432 cases), non-antiviral treatment (1094 cases), high HBV DNA levels (3831 cases), HBeAg positive (1592 cases), family histories of HCC (685 cases) and gender (male) (3085 cases). The details of all of the studies evaluated in this meta-analysis are shown in Table 1 [9,13,14,15,16,17,18,19,20,21,22,23,24,25,26,27,28,29,30,31,32,33,34,35,36,37,38]. Twenty-seven studies fulfilled the requirements and these included 14,061 objects (see Table 1).

The available sample size of each study varied greatly, ranging from 80 to 11,893 objects. The mean age was greatly variable and the percentage of females also differed greatly (see Table 1). 

### 3.3. Effects of Related Factors on the Development of HCC

**Figure 2:**
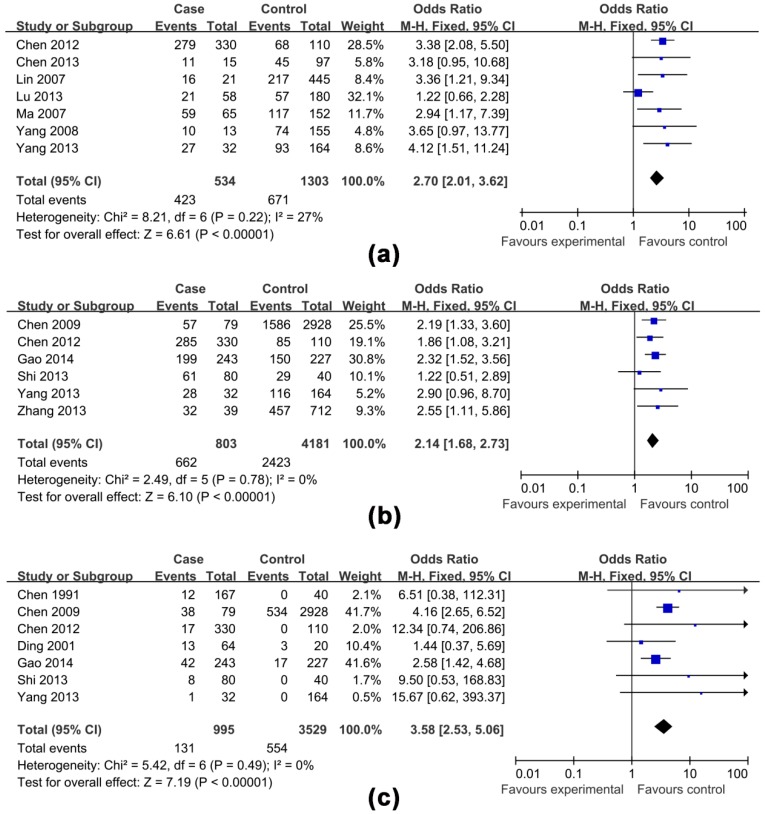
Effects of possible risk factors in chronic HBV-infected patients: (**a**) non-antiviral treatment; (**b**) male gender; and (**c**) a family history of HCC.

**Figure 3:**
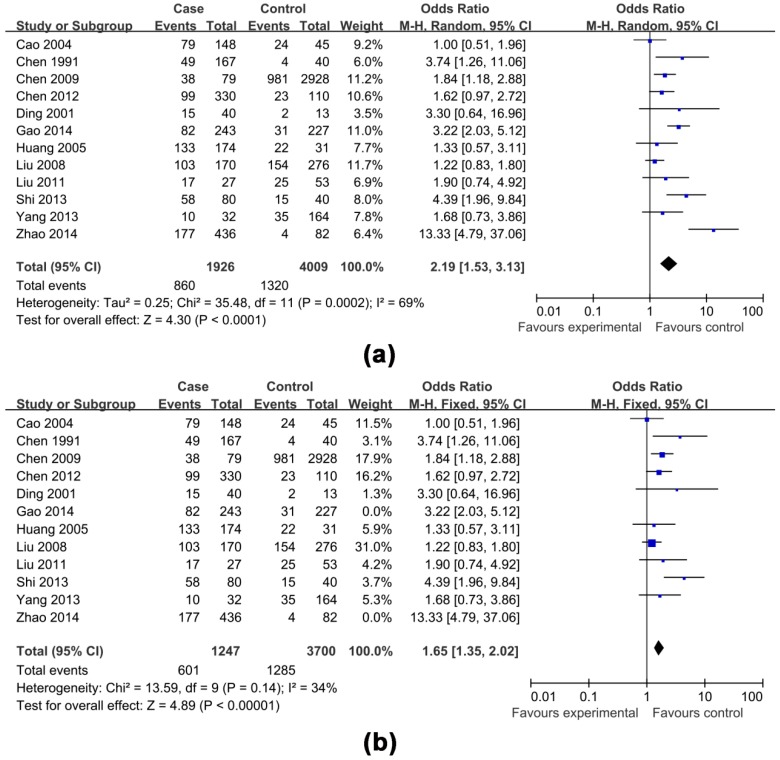
Effects of possible risk factors in chronic HBV-infected patients: (**a**) drinking alcohol; and (**b**) drinking alcohol adjusted.

**Figure 4:**
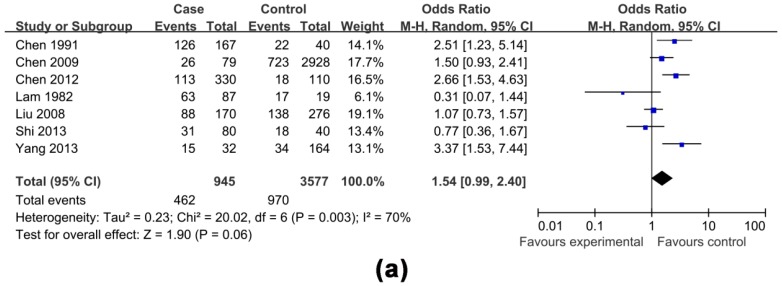

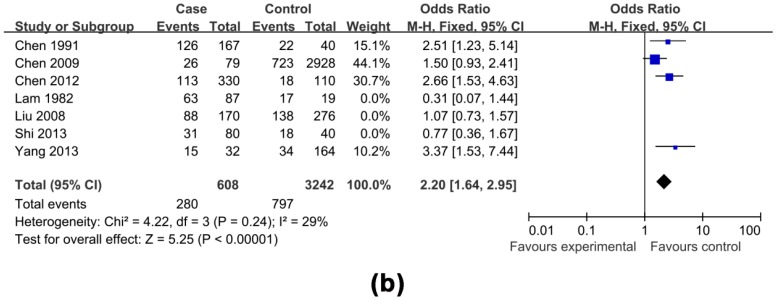
Effects of possible risk factors in chronic HBV-infected patients: (**a**) smoking; and (**b**) smoking adjusted.

**Figure 5:**
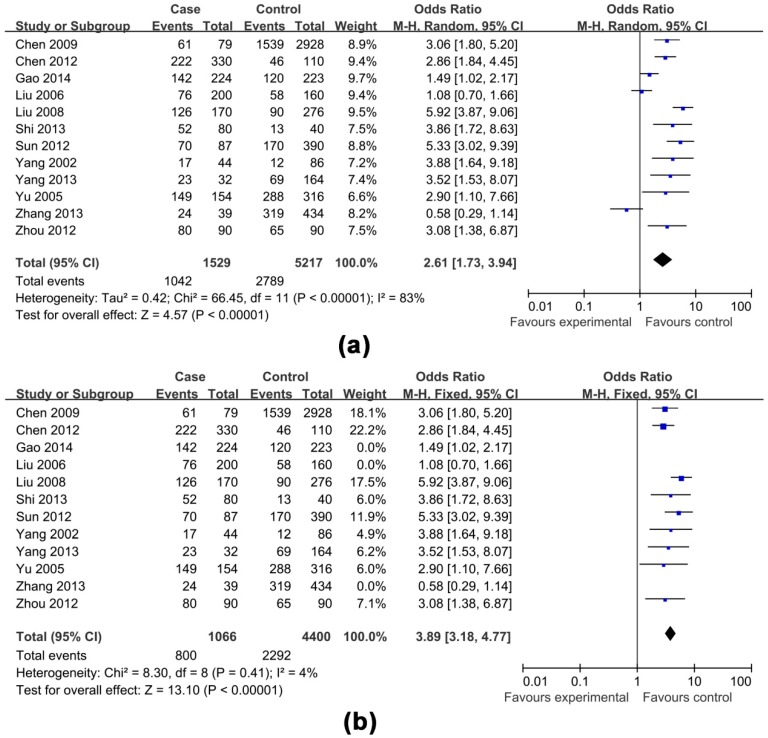
Effects of possible risk factors in chronic HBV-infected patients: (**a**) high HBV DNA levels; and (**b**) high HBV DNA levels adjusted.

**Figure 6:**
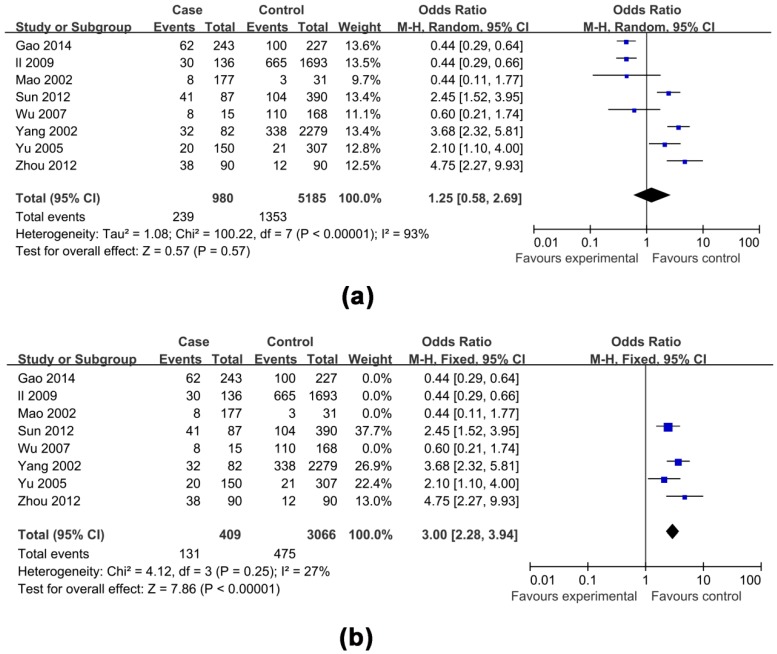
Effects of possible risk factors in chronic HBV-infected patients: (**a**) HBeAg positive; and (**b**) HBeAg positive adjusted.

The effects of non-antiviral treatment (seven studies, 1837 research objects), alcohol consumption (twelve studies, 5935 research objects), smoking (seven studies, 4522 research objects), high HBV DNA levels (twelve studies, 6746 research objects), HBeAg positive (eight studies, 6165 research objects), family histories of HCC (seven studies, 4524 research objects) and gender (male) (six studies, 4984 research objects) on the development of HCC were investigated in this analysis and the results are shown in Figure 2, Figure 3, Figure 4, Figure 5 and Figure 6, respectively.

The pooled OR with 95% CI for the seven factors investigated were: non-antiviral treatment 2.70 (2.01, 3.62), high HBV DNA levels 2.61 (1.73, 3.94), alcohol consumption 2.19 (1.53, 3.13), a family history of HCC 3.58 (2.53, 5.06), male 2.14 (1.68, 2.73), smoking 1.54 (0.99, 2.40) and HBeAg positive 1.25 (0.58, 2.69), respectively.

The heterogeneity test indicated that the variation of study-specific OR for alcohol consumption, smoking, high HBV DNA levels, HBeAg positive, were statistically significant (*p* < 0.10), and the random effect method was therefore used to pool the results for these factors. The heterogeneity test indicated that the variation of study-specific OR were not statistically significant (*p* > 0.10), and the fixed effect method was therefore used to pool the results for the other factors, and the conclusions remained largely unchanged when the random effect method was used. The results of statistical analysis and calculation are shown in Table 2.

### 3.4. Sensitivity Analysis and Publication Bias

**Figure 7:**
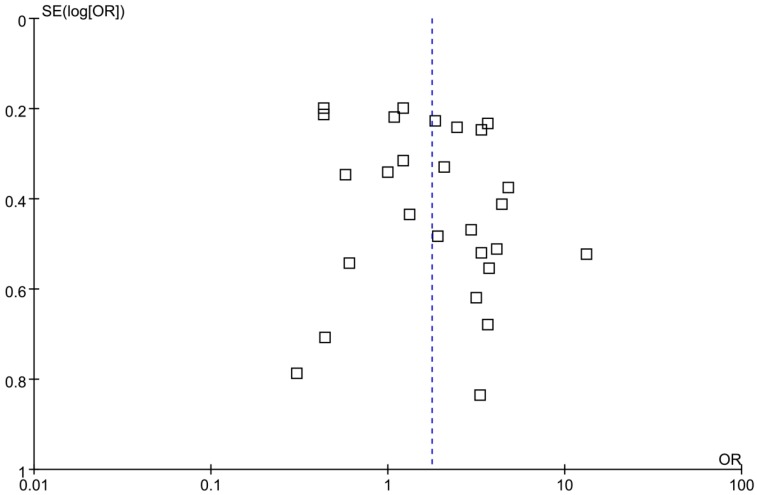
A funnel plot for publication bias.

Considering the enormous heterogeneity in some subgroups, we used sensitivity analysis to identify possible heterogeneous records from eligible studies. After omitting the selected studies, the study-specific OR results with publication bias and lower degree of heterogeneity are shown in Table 3, and the effects of four factors on the development of HCC are shown in Figure 3, Figure 4, Figure 5 and Figure 6, respectively. A funnel plot for publication bias is shown in Figure 7.

## 4. Discussion

The development of HCC is a complex process that goes from liver damage to liver cell transformation, involving multiple risk factors. However, most of these factors can be prevented to decrease the incidence of HCC [48,49,50]. In this study, we attempted to carry out a comprehensive analysis of HCC risk factors. Our meta-analysis collated all of the available literature to determine the association between the main risk factors and HCC in the chronic HBV-infected Chinese [46,47].

The results of this meta-analysis showed that, compared with not having the corresponding factor, the chronic HBV-infected Chinese with high HBV DNA levels and non-antiviral treatment have nearly treble the risk of HCC development. These findings also were confirmed by other studies [24,25,32]. This indicated that antiviral treatment could greatly decrease the number of HCC development in the chronic HBV-infected Chinese.

The results of this meta-analysis also showed that, compared with not having the corresponding factor, alcohol consumption, a family history of HCC and gender (male) have 2–4 times the risk of HCC development. The data do lead us to believe that controlling alcohol consumption might lead to decreases in HCC for some chronically infected Chinese. Since the proportion of the population with one or more of these risk factors is high in China, professional health education should be enhanced to promote cognitive and behavioral changes to reduce these harmful factors in society, and thus speeding up the process of preventing and controlling HCC. There is a high prevalence of non-antiviral treatment and alcohol drinking [14,29,30,38,51,52]. Many patients cannot afford treatment and patients are not sure how to manage HBV. Furthermore, many providers do not prescribe the long-term ill effect of drug and drug resistance or give attention to systemic issues, even though they affect the therapeutic efficacy of chronic hepatitis B. In view of the above-mentioned facts, effective interventions for the two factors should be tackled first.

In addition, this study showed that HBeAg positivity did not affect HCC development, and the results were different from those observed in previously reported [20,33]. A possible reason for this was that some cases had received antiviral treatment in four studies [13,22,25,35]. After omitting these four studies, HBeAg positivity was associated with an increase in the risk of HBV-related HCC. Confirmatory research should be carried out to ascertain the real association between HBeAg positivity and HCC. 

This study also showed that smoking did not affect HCC development, and the findings were also different from those observed in previously reported research [53]. After omitting three retrospective studies, smoking was associated with an increase in the risk of HBV-related HCC [23,24,36]. Confirmatory studies with different environment and gene background should be carried out to ascertain the real association between smoking and HBV-related HCC.

It has previously been reported that age was a risk factor for HBV-related HCC development [54]; however, in the literature used in our study, age was indicated as the median age, mean age or age range. In fact, individual data (e.g., patient-level data) were not available in the majority of cases; thus, it was impossible to analyze the association between age and HBV-related HCC development.

Heterogeneity in the variation of study-specific OR for high HBV DNA levels and positive HBeAg was determined. One possible reason was that some cases had received antiviral treatment in three studies and four studies [13,21,22,25,34,35], respectively. These studies were excluded, and heterogeneity in the variation of study-specific OR for the two factors was not found. In addition, heterogeneity was found in the variation of study-specific OR for alcohol consumption and smoking. One possible reason is that there was information bias in two retrospective studies and three retrospective studies [23,24,25,26,36], respectively. These studies were excluded, and heterogeneity in the variation of study-specific OR for these two factors was not found.

This study has several limitations: (1) Some observational studies, retrospective studies and nonrandomized designs are susceptible to various biases such as inappropriate selection of subjects. These biases could have influenced slightly the internal and external validity of this study; (2) Although 27 eligible studies were included in our analysis, the sample for subgroup analysis was limited which could have affected the results; (3) Baseline data of all eligible studies such as observation time did not match well, and this could have underestimated the results; (4) Due to the low possible publication rate for negative studies, publication bias existed in some subgroup analyses of possible risk; (5) As the development of HBV-related HCC may be caused by multiple factors simultaneously, the interaction of factors may have contributed to the results. Due to data limitations, this article did not analyze this interaction.

## 5. Conclusions

Our meta-analysis supports that high HBV DNA levels, non-antiviral treatment, alcohol consumption, a family history of HCC and male gender contributed to the risk of hepatocellular carcinoma in chronic HBV-infected Chinese from currently available evidence. Given the high prevalence of non-antiviral treatment and alcohol drinking, behavior interventions for these two factors should be tackled first.

## Figures and Tables

**Table 1:** Characteristics of the studies.

Study	Region	Study Type	Participants Category (Case/Control)	Risk Factors	Sample Size (n)	Age (Years)	Male/Female
[17]	Taixing	case-control study	HCC/nature people	alcohol consumption	208, 208	52 (22–93)	170/38
[15]	Taiwan	case-control study	HCC/nature people	alcohol consumption, smoking, family histories of HCC	200, 200	*p* *	*p* *
[32]	Longan	prospective study	choric HBsAg carries	alcohol consumption, smoking, HBV DNA levels, family histories of HCC, sex	3007	33–55	1643/1364
[38]	Jiangsu, Qidong	case-control study	HBV-related HCC/LC	alcohol consumption, smoking, antiviral treatment, HBV DNA levels, family histories of HCC, sex	330, 110	52.4 ± 10.6, 51.4 ± 10.6	285/45, 85/25
[29]	Chongqing	prospective study	CHB	antiviral treatment	112	33.7 ± 9.1, 32.4 ± 8.7	59/53
[18]	Taixing	case-control study	HCC/nature people	alcohol consumption, family histories of HCC	88, 88	47.5 (22–79)	71/17
[25]	Qingdao	case-control study	HBV-related HCC/CHB	alcohol consumption, HBV DNA levels, HBeAg positive, family histories of HCC, sex	243, 227	57 (51, 63), 54 (50, 59)	199/44, 150/77
[39]	Jiangsu, Huaian	case-control study	HCC/digestion tract cancer not HCC	alcohol consumption	219, 219	53 (26–78), 50.5 (28–75)	153/66, 151/68
[23]	Hong kong	case-control study	HBV-related HCC/trauma patients	smoking	150, 36	*p* *	*p* *
[22]	Kunming	retrospective study	HBeAg(+)CHB/HBeAg(−)CHB	HBeAg positive	1829	42.07 ± 14.22	1471/358,
[27]	Taiwan	prospective study	HBeAg(+)CHB/HBeAg(+)CHB	antiviral treatment	233, 233	32 ± 7, 31 ± 8	219/14, 219/14
[21]	Taiwan	case-control study	HCC/choric HBV carries	HBV DNA levels	200, 160	54.0 ± 12.3, 32 ± 10.0	167/200, 103/160
[24]	Jiangsu, Qidong	case-control study	choric HBsAg carries/choric HBsAg carries	alcohol consumption, smoking, HBV DNA levels	170, 276	>20	170/0, 276/0
[31]	Hubei	prospective study	CHB	alcohol consumption	80	49.3 ± 13.2	80/0
[30]	Urumqi	prospective study	HBV-related LC	antiviral treatment	238	45.7 ± 11.3	168/70
[14]	Beijing	prospective study	choric HBV-related LC	antiviral treatment	217	54 ± 10	157/60
[35]	Hunan	case-control study	HCC/digestive malignancy not HCC	HBeAg positive	211, 220	13–76	192/19, -
[36]	Shanghai	case-control study	HBV-related HCC/HBV-related LC	alcohol consumption, smoking, HBV-DNA levels, family histories of HCC, sex	80, 40	48.72 ± 14.15, 50.14 ± 16.32	61/19, 29/11
[28]	Jiangsu, Qidong	prospective study	choric HBsAg carries and not choric HBsAg carries	HBV DNA levels, HBeAg positive	477, 477	43.9 ± 9.8, 43.6 ± 10.3	392/85, 392/85
[13]	Chongqing	prospective study	CHB	HBeAg positive	183	31.9 ± 8, 31.1 ± 7.2	52/13, 100/18
[19]	Taiwan	prospective study	nature people	HBV DNA levels, HBeAg positive	11,893	30–65	11,893/0
[16]	Shenyang	prospective study	Decompensated liver disease	antiviral treatment	84, 84	26–78	59/25, 65/19
[37]	Taizhou, Linhai	prospective study	HBV-related LC	alcohol consumption, smoking, antiviral treatment, HBV DNA levels, family histories of HCC, sex	196	47.37 ± 5.62, 45.08 ± 6.83	144/52
[20]	Taiwan	prospective study	choric HBsAg carries	HBV DNA levels, HBeAg positive	4841	>30	4841/0
[34]	Wuhan, Xiaogan	prospective study	CHB	HBV DNA levels, sex	751	51 ± 4.6	489/262
[26]	Guiyang	case-control study	HCC/Cancer not HCC	alcohol consumption	762, 798	51 (22–89), 48 (19–86)	602/160, 458/340
[33]	Nanjing	case-control study	choric HBsAg carries/choric HBsAg carries	HBV DNA levels, HBeAg positive	90, 90	52.53 ± 10.27, 52.53 ± 10.23	78/12, 78/12

* *p*, the difference of corresponding variable between case and control wasn’t significant (*p* > 0.05); Abbreviations: HCC, hepatocellular carcinoma; CHB, chronic hepatitis B; LC, liver cirrhosis.

**Table 2:** The subgroup characteristics of possible risk factors associated with HCC in HBsAg-positive Chinese population.

Risk Factors	No. of Studies (Cases/Controls)	OR			M	Heterogeneity	P*_H_*	I^2^ (%)	Egger’s Test	*p*	Begg’s Test	*p*
		OR (95% CI)	Z	*P_OR_*		X2			*t*		Z	
subgroup analyses by non-antiviral treatment	7 (534/1303)	2.70 (2.01, 3.62)	6.61	<0.05	F	8.21	>0.1	0.27	0.65	>0.1	0	>0.1
subgroup analyses by alcohol consumption	12 (1926/4009)	2.19 (1.53, 3.13)	4.3	<0.05	R	35.48	<0.1	0.69	1.48	>0.1	1.3	>0.1
subgroup analyses by smoking	7 (945/3577)	1.54 (0.99, 2.40)	1.9	>0.05	R	20.02	<0.1	70%	−0.04	>0.1	0	>0.1
subgroup analyses by high HBV DNA levels	12 (1529/5217)	2.61 (1.73, 3.94)	4.57	<0.05	R	66.45	<0.1	0.83	0.54	>0.1	0.21	>0.1
subgroup analyses by HBeAg positive	8 (980/5185)	1.25 (0.58, 2.69)	0.57	>0.05	R	100.22	<0.1	0.93	0.45	>0.1	0.12	>0.1
subgroup analyses by family histories of HCC	7 (995/3529)	3.58 (2.53, 5.06)	7.19	<0.05	F	5.42	>0.1	0	0.81	>0.1	0.3	>0.1
subgroup analyses by male	6 (803/4181)	2.14 (1.68, 2.73)	6.1	<0.05	F	2.49	>0.1	0	−0.27	>0.1	0	>0.1

The three factor = non-antiviral treatment, alcohol consumption and depression. Abbreviations: HCC = hepatocellular carcinoma; OR = Odds ratio; CI = confidence intervals; M = model; F = fixed-effect Model; R = random-effect model.

**Table 3:** The adjusted subgroup characteristics of possible risk factors associated with HCC in HBsAg-positive Chinese population.

Risk Factors	No. of Studies (Cases/Controls)	OR			M	Heterogeneity	P*_H_*	I^2^ (%)	Egger’s Test	*p*	Begg’s Test	*p*	Omitted Reference
		OR (95% CI)	Z	*P_OR_*		X2			*t*		Z		
subgroup analyses by high HBV DNA levels	9 (1066/4400)	3.89 (3.18, 4.77)	13.10	<0.05	F	8.30	>0.1	4%	−0.76	>0.1	−0.1	>0.1	[21,25,34]
subgroup analyses by alcohol consumption	10 (1247/3700)	1.65 (1.35, 2.02)	4.89	<0.05	F	13.59	>0.1	34%	1.73	>0.1	1.43	>0.1	[25,26]
subgroup analyses by HBeAg positive	4 (409/3066)	3.00 (2.28, 3.94)	7.86	<0.05	F	4.12	>0.1	27%	0.19	>0.1	−0.34	>0.1	[13,22,25,35]
subgroup analyses by smoking	4 (608/3242)	2.20 (1.64, 2.95)	5.25	<0.05	F	4.22	>0.1	29%	2.03	>0.1	1.02	>0.1	[23,24,36]

Abbreviations: HCC = hepatocellular carcinoma; OR = Odds ratio; CI = confidence intervals; M = model; F = fixed-effect model.

## Abbreviations

The following abbreviations are used in this manuscript:

HCC	hepatocellular carcinoma
OR	odds ratios
CI	confidence intervals
HBV	hepatitis B virus
HBsAg	hepatitis B virus surface antigen
HBeAg	hepatitis B e antigen
CNKI	National Knowledge Infrastructure
VIP	VIP database
Wanfang	Wanfang data
M	model
F	fixed-effect model
R	random-effect model

## References

[1] Gomaa A.I., Khan S.A., Toledano M.B., Waked I., Taylor-Robinson S.D. (2008). Hepatocellular carcinoma: Epidemiology, risk factors and pathogenesis. World J. Gastroenterol..

[2] Parkin D.M., Bray F., Ferlay J., Pisani P. (2005). Global cancer statistics, 2002. CA Cancer J. Clin..

[3] Bosch F.X., Ribes J., Cléries R., Díaz M. (2005). Epidemiology of hepatocellular carcinoma. Clin. Liver Dis..

[4] Zhang S., Zheng R., Li N., Zeng H., Dai Z., Zou X., Chen W. (2012). Analysis and prediction of liver cancer incidence in China. Zhonghua Yu Fang Yi Xue Za Zhi.

[5] Stewart B.W., Wild C. (2014). World Cancer Report 2014.

[6] Zhang Y.Z., Li X., Zhou W.J., Niu W.M., Liu P., Li Y. (2012). Study on microcystins-LR pollution situation of Taihu rim. J. Food Saf. Q..

[7] Shandong Lu Hua Group, Co., Ltd. (2008). Squeezing methods of oil production and technology of getting rid of aflatoxin. Food Saf. Guide.

[8] Zhang W.H. (1995). The role of Chinese herbal medicine in the elimination of aflatoxin. Food Storage.

[9] Huang G.Y., Ma X.G., Wang C.C. (2005). Heavy alcohol consumption can enhance risk of hepatocellular carcinoma associated with hepatitis B virus infection. J. Oncol..

[10] Liang X., Bi S., Yang W., Wang L., Cui G., Cui F., Zhang Y., Liu J., Gong X., Chen Y. (2013). Reprint of: Epidemiological serosurvey of Hepatitis B in China—Declining HBV prevalence due to Hepatitis B vaccination. Vaccine.

[11] Liang X., Bi S., Yang W., Wang L., Cui G., Cui F., Zhang Y., Liu J., Gong X., Chen Y. (2009). Evaluation of the impact of hepatitis B vaccination among children born during 1992–2005 in China. J. Infect. Dis..

[12] Chang M.H. (2014). Prevention of hepatitis B virus infection and liver cancer. Viruses and Human Cancer.

[13] Wu G.C., Zhou W.P., Zhao Y.R., Guo S.H., Huang A.L., Reng H., Zhang D.F. (2007). A study on the long-term outcome of hepatitis B e antigen-negative chronic hepatitis B compared with that of hepatitis B e antigen-positive chronic hepatitis B. Chin. J. Infect. Dis..

[14] Ma H., Guo F., Wei L., Sun Y., Wang H. (2007). The prospective study of the clinical features and outcome of HBeAg-negative and HBeAg-positive cirrhosis in patients with chronic type B hepatitis. Chin. Med. J..

[15] Chen C.J., Liang K.Y., Chang A.S., Chang Y.C., Lu S.N., Liaw Y.F., Chang W.Y., Sheen M.C., Lin T.M. (1991). Effects of hepatitis B virus, alcohol drinking, cigarette smoking and familial tendency on hepatocellular carcinoma. Hepatology.

[16] Yang F., Wu Y.H., Liu D.Y., Li W.L., Wei N. (2008). The effect of lamivudine treatment on the prognosis of patients with hepatitis B related decompensative liver diseases. Chin. J. Infect. Dis..

[17] Cao H.X., Ding J.H., Wu J.Z. (2004). The Impact of Alcohol Drinking Habit and HBsAg on the Risk of Hepatocellular Carcinoma in Taixing City. Bull. Chin. Cancer.

[18] Ding J.H., Li S.P., Wu J.Z., Gao C.M., Zhou J.N., Su P., Liu Y.T., Zang Y., Zhou X.F., Ding B.G. (2001). The Study on the Interactions between HBsAg and Various Risk Factors in Primary Hepatocellular Carcinoma in Taixing Area. Bull. Chin. Cancer.

[19] Geier A., Gartung C., Dietrich C.G., Yang H. (2002). Hepatitis B e antigen and the risk of hepatocellular carcinoma. N. Engl. J. Med..

[20] Yu M.W., Yeh S.H., Chen P.J., Liaw Y.F., Lin C.L., Liu C.J., Shi W.L., Kao J.H., Chen D.S., Chen C.J. (2005). Hepatitis B virus genotype and DNA level and hepatocellular carcinoma: A prospective study in men. J. Natl. Cancer Inst..

[21] Liu C.J., Chen B.F., Chen P.J., Lai M.Y., Huang W.L., Kao J.H., Chen D.S. (2006). Role of hepatitis B viral load and basal core promoter mutation in hepatocellular carcinoma in hepatitis B carriers. J. Infect. Dis..

[22] Li W., He G.Q., Duan L.F., Wei J., Yang W.B. (2009). Clinical characteristics of 1829 cases of HBeAg positive chronic hepatitis B. J. Kunming Med. Univ..

[23] Lam K.C., Yu M.C., Leung J.W., Henderson B.E. (1982). Hepatitis B virus and cigarette smoking: Risk factors for hepatocellular carcinoma in Hong Kong. Cancer Res..

[24] Liu T.T., Fang Y., Xiong H., Chen T.Y., Ni Z.P., Luo J.F., Zhao N.Q., Shen X.Z. (2008). A case-control study of the relationship between hepatitis B virus DNA level and risk of hepatocellular carcinoma in Qidong, China. World J. Gastroenterol..

[25] Gao R., Liu Z.J., Chen F., Gao L., Jiang C.W. (2014). Mutiviate Regression Analysis of Risk Factors for HBV-related Primary Liver Cancer. J. Clin. Hepatol..

[26] Zhao X.K., Zhang Q., Chen S.S., Tan J.W., Wang W.Z., Chen M.L. (2014). A case-control study on risk factors of primary hepatocellular cancer in Guizhou. Chongqing Med..

[27] Lin S.M., Yu M.L., Lee C.M., Chien R.N., Sheen I., Chu C.M., Liaw Y.F. (2007). Interferon therapy in HBeAg positive chronic hepatitis reduces progression to cirrhosis and hepatocellular carcinoma. J. Hepatol..

[28] Sun Y., Chen T.Y., Lu P.X., Wu Y., Zhang Q.N., Qian G.S., Tu H. (2012). Relationship between serum hepatitis B virus DNA load and hepatocellular carcinoma in Qidong, China: A cohort follow-up study of 14 years. Natl. Med. J. China.

[29] Chen Y., Sun W.J., Han X.Y. (2013). Relationship analysis between hepoatocelluar carcinoma incidence and hepatitis B e antigen expression in chronic hepatitis B patients with long-term treatment of nucleoside analogues therapy. Chin. J. Postgrad. Med..

[30] Lu X.B., Sha N.Y., Zhen Z.R., Li G., Zhang Z.G., Zhang Y.X. (2013). The Effects of Long-term Antiviral Therapy on the Prognosis of patients with Hepatitis B-induced Cirrhosis. Chin. Hepatol..

[31] Liu Y.W., Guo X.P. (2011). Priming effects of alcohol on the risk factors of hepatocellular carcinoma in chronic hepatitis B. Chin. Hepatol..

[32] Chen Q.Y., Dong B.Q., Yang J.Y., Wei S.C., Fang K.X., Wang X.Y. (2009). A prospective study of the relationship between serum hepatitis B virus DNA and the risk of primary liver cancer. Chin. J. Hepatol..

[33] Zhou J.Y., Zhang L., Li L., Gu G.Y., Zhou Y.H., Chen J.H. (2012). High hepatitis B virus load is associated with hepatocellular carcinomas development in Chinese chronic hepatitis B patients: A case control study. Virol. J..

[34] Zhang Z.B., Jiang Y.A. (2013). Patients with chronic hepatitis B liver factors occurring nucleoside analogues 10 antiretroviral therapy. J. Clin. Hepatol..

[35] Mao J.K., Li H.Y., Zhou Y. (2002). A serological investigation on relationship between the infection rate of HBV and the PHC. Pract. Prev. Med..

[36] Shi C.C., Xue F., Sun Y.F. (2013). Evaluation of Risk Factors of Primary Hepatic Carcinoma Due to Hepatitis B Cirrhosis. Pract. J. Cancer.

[37] Yang X.M., Zhang W., Zheng H.B. (2013). Analysis of risk factors for hepatitis B liver cirrhosis complicated with primary cancer. China Mod. Dr..

[38] Chen P., Li J., Su F., Li J.B., Cheng F. (2012). Risk factors for hepatocellular carcinomas in patients with liver cirrhosis. Acta Univ. Anhui.

[39] Truong B.X., Seo Y., Kato M., Hamano K., Ninomiya T., Katayama M., Kato H., Yano Y., Hayashi Y., Kasuga M. (2005). Long-term follow-up of Japanese patients with chronic hepatitis B treated with interferon-α. Int. J. Mol. Med..

[40] Tangkijvanich P., Thong-ngam D., Mahachai V., Kladchareon N., Suwangool P., Kullavanijaya P. (2001). Long-term effect of interferon therapy on incidence of cirrhosis and hepatocellular carcinoma in Thai patients with chronic hepatitis B. Southeast Asian J. Trop Med. Public Health.

[41] Key Documents. http://www.prisma-statement.org/.

[42] Liu Y., Chang C.-C., Marsh G.M., Wu F. (2012). Population attributable risk of aflatoxin-related liver cancer: Systematic review and meta-analysis. Eur. J. Cancer.

[43] Chinese Anti-Cancer Associationand the Professional Committee of Liver Cancer Clinical Oncology Collaboration Committee, China HCC Medical Association Hepatology Study Group (2009). Diagnosis and treatment of primary liver cancer experts from standardization knowledge. J. Clin. Oncol..

[44] Chinese Society of Hepatology and Chinese Society of Infectious Diseases, Chinese Medical Association (2011). The guideline of prevention and treatment for chronic hepatitis B (2010 version). Zhonghua Liu Xing Bing Xue Za Zhi.

[45] Dersimonian R., Laird N. (1986). Meta-analysis in clinical trials. Controll. Clin. Trials.

[46] Egger M., Smith G.D., Schneider M., Minder C. (1997). Bias in meta-analysis detected by a simple, graphical test. BMJ.

[47] Sterne J.A., Egger M. (2001). Funnel plots for detecting bias in meta-analysis: Guidelines on choice of axis. J. Clin. Epidemiol..

[48] Wild C.P., Hall A.J. (2000). Primary prevention of hepatocellular carcinoma in developing countries. Mutat. Res./Rev. Mutat. Res..

[49] Monto A., Wright T.L. (2001). The epidemiology and prevention of hepatocellular carcinoma. Seminars in Oncology.

[50] Anwar W.A., Khaled H.M., Amra H.A., El-Nezami H., Loffredo C.A. (2008). Changing pattern of hepatocellular carcinoma (HCC) and its risk factors in Egypt: Possibilities for prevention. Mutat. Res./Rev. Mutat. Res..

[51] Zang N., Wu J.Z., Chen W.Q., Wu J.L., Ning Q.Y., Deng Y.M., Wei Y.H., Hu D.F., Li L.L., Huang A.C. (2011). Comparative Analysis of Aetiology Clustering of Hepatocellular Carcinoma between Two Families with High Mortality of Hepatoceilular Carcinoma Discovered Recently in Guangxi Region. Tumor.

[52] Lu L., Wang X. (2008). Drug addiction in China. Ann. N. Y. Acad. Sci..

[53] Jiang Y., Qin C.Y. The Association of Cigarette Smoking with PDCD4 in Hepatocellular Carcinoma. http://kns.chkd.cnki.net/kcms/detail/detail.aspx?recid=&FileName=2009248620.nh&DbName=CDMH9911&DbCode=CDMH&uid=WEEvREcwSlJHSldTTEYzUk1zUTdMT2dib1lwNzdyVy9yOFZvVUIxeXRnQko5Zm5XM1UxRUNobk1lZzFOc0ltRXpBPT0=$9A4hF_YAuvQ5obgVAqNKPCYcEjKensW4IQMovwHtwkF4VYPoHbKxJw!!.

[54] But D.Y., Lai C.L., Yuen M.F. (2008). Natural history of hepatitis-related hepatocellular carcinoma. World J. Gastroenterol..

